# The chemical composition of mineral trioxide aggregate

**DOI:** 10.4103/0972-0707.48834

**Published:** 2008

**Authors:** Josette Camilleri

**Affiliations:** Department of Building and Civil Engineering, Faculty of Architecture and Civil Engineering, Department of Dental Surgery, Faculty of Dental Surgery, University of Malta, Malta

**Keywords:** Mineral trioxide aggregate, Portland cement, chemical composition

## Abstract

Mineral trioxide aggregate (MTA) is composed of Portland cement, with 4:1 addition of bismuth oxide added so that the material can be detected on a radiograph. The cement is made up of calcium, silicon and aluminium. The main constituent phases are tricalcium and dicalcium silicate and tricalcium aluminate. There are two commercial forms of MTA, namely the grey and the white. The difference between the grey and the white materials is the presence of iron in the grey material, which makes up the phase tetracalcium alumino-ferrite. This phase is absent in white MTA. Hydration of MTA occurs in two stages. The initial reaction between tricalcium aluminate and water in the presence of calcium sulphate results in the production of ettringite. Tricalcium and dicalcium silicate react with water to produce calcium silicate hydrate and calcium hydroxide, which is leached out of the cement with time.

## INTRODUCTION

Mineral trioxide aggregate (MTA) was developed at Loma Linda University, in the 1990s, as a root-end filling material. It is used primarily to seal lateral root perforations[1,2] and as a root-end filling material.[3–6] The use of MTA as a root-end filling material was identified because the material is hydraulic and sets in the presence of water. Mineral trioxide aggregate (MTA) received acceptance by the US Federal Drug Administration and became commercially available as ProRoot MTA (Tulsa Dental Products, Tulsa, OK, USA). Until recently, two commercial forms of MTA were available (ProRoot MTA), in either the grey or white forms. Recently MTA-Angelus (Angelus Soluções Odontológicas, Londrina, Brazil) has become available.

## MTA PATENT

The MTA patent[7] stated that ‘MTA consists of 50-75% (wt) calcium oxide and 15-25% silicon dioxide. These two components together comprise 70-95% of the cement. When these raw materials are blended, they produce tricalcium silicate, dicalcium silicate, tricalcium aluminate, and tetracalcium aluminoferrite. On addition of water, the cement hydrates, forming silicate hydrate gel.’ Also ‘MTA is Type 1 Portland cement (American Society for Testing Materials), with a fineness (Blaine number) in the range of 4500-4600 cm^2^/g. A radiopacifier (bismuth oxide) is added to the cement for dental radiological diagnosis.’[7]

## GREY AND WHITE MTA

Commercial MTA exists in both grey and white forms (Dentsply, Tulsa Dental Products, Tulsa, OK, USA). Recently, a Brazilian company produced MTA-Angelus (Angelus Soluções Odontológicas, Londrina, Brazil). The difference between the grey MTA and the white MTA has been reported to be in the lack of iron in the white version.[8,9]

## MTA AND PORTLAND CEMENT

The similarity of MTA with Portland cement was reported only in 2000.[10] Further studies comparing white MTA (White MTA, Dentsply, Tulsa Dental Products, Tulsa, OK, USA) with white Portland cement showed the cements to have similar constituent elements, except for the bismuth oxide in the MTA.[8,9,11,12] Similar results were obtained when comparing MTA Angelus with Portland cement.[13] Both MTA and Portland cement were biocompatible, as the composition of both materials was similar.[14] Further investigation of both Portland cement and MTA showed some differences between the materials. Scanning electron microscopy of the polished sections of both MTA and Portland cement showed that the aluminate phase normally present in Portland cement was scarce in MTA.[15] The MTA had a lower level of tricalcium silicate and a higher level of dicalcium silicate, when compared to white Portland cement. There was no tricalcium aluminate present in the MTA, suggesting that the material was not prepared in a rotary kiln, as is customary for the manufacture of Portland cement. Less calcium sulphate was found in MTA. Portland cement had a total of 4.9% calcium sulphate present as di- and hemi-hydrate and anhydrite. Mineral trioxide aggregate (MTA) had only 2.2% calcium sulphate and the di-hydrate was absent.[16]

## CHEMICAL COMPOSITION OF MTA POWDER

Energy dispersive analysis with X-ray (EDAX) demonstrated that MTA was composed of calcium, silicon, aluminium and bismuth.[8,9] Scanning electron microscopy (SEM) of the polished section showed that MTA consisted of particles ranging between <1 *μ*m to approximately 30 *μ*m in size, containing impure tricalcium silicate and impure dicalcium silicate. Particles (10 *μ*m - 30 *μ*m) of bismuth oxide were numerous. Scattered particles of limestone or hydrated lime, gypsum and potassium aluminium silicate were also present.

Deficiency of alumina was seen in MTA.[15] An X-ray diffraction (XRD) analysis showed the presence of a completely crystalline material composed mainly of tricalcium and dicalcium silicate and bismuth oxide.[8,9] Precise quantitative analysis of MTA, using Rietveld XRD, showed that MTA was composed of tricalcium and dicalcium silicate, tricalcium aluminate and calcium sulphate, which was present in the hemi-hydrate and anhydrite form. In addition, MTA contained 21.6% bismuth oxide.[16] This is similar to what was reported in the MTA patent.[3]

## HYDRATION OF MTA

The hydration of MTA has been reported to consist of two separate reactions. The initial reaction was between tricalcium aluminate and water, which, in the presence of gypsum found in small quantities in MTA, resulted in the production of ettringite, which later formed monosulphate, once the gypsum was depleted. The low levels of alumina reported in MTA affected the production of ettringite and monosulphate, usually formed on hydration of Portland cement.[15] The main reaction between the tricalcium and dicalcium silicate and water resulted in the production of calcium silicate hydrate gel, which is poorly crystalline, and calcium hydroxide.[15] Set MTA was composed of numerous residual un-hydrated cement grains, which had a dense rim of hydration product, made up of pure calcium silicate hydrate. There was very little ettringite or monosulphate present. Un-reacted bismuth oxide particles and calcium hydroxide were also detected. The calcium silicate hydrate had taken up bismuth, which replaced the silica in the calcium silicate hydrate structure.[15]

Bismuth oxide, added to enhance the radio-opacity of MTA, was reported to be present only in 8.4% level in set MTA, as against the 21.6% in the unset material.[16] The bismuth formed a part of the structure of the calcium silicate hydrate gel and also affected the precipitation of calcium hydroxide in the hydrated paste.[15] Both bismuth and calcium were leached out from MTA. The calcium leached out decreased over a five-week period, while the bismuth oxide levels increased.[16]

The production of calcium hydroxide by MTA would explain the similar mode of tissue reaction to MTA and calcium hydroxide reported previously.[17,18] It has been reported that MTA released calcium ions[19,20] and promoted an alkaline pH.[21,22] MTA has been shown to leach calcium ions several days after the initiation of hydration and setting of the material.[16] These calcium ions diffuse through the defects in the dentin in root canals filled with MTA, and the concentration increases with time.[23] The physicochemical basis for the biological properties of MTA had recently been attributed to the production of hydroxyapatite, when the calcium ions released by the MTA came into contact with tissue fluid.[24,25] When in contact with tissue fluid, an amorphous calcium phosphate phase initially formed, which later transformed to an apatite phase, with the latter consisting of calcium-deficient, poorly crystalline, B-type carbonated apatite crystallites. Amorphous calcium phosphate is a key intermediate that precedes biological apatite formation in skeletal calcification.[26]
